# Supplementary material for: Edwardsiella tarda TraT is an anti-complement factor and a cellular infection promoter
Source: Commun Biol. 2022 Jun 29;5:637. doi: 10.1038/s42003-022-03587-3 (PMC9243006; doi:10.1038/s42003-022-03587-3)
Supplement: Supplementary file 2 — Supplementary Information [file 42003_2022_3587_MOESM2_ESM.pdf]

Supplementary Figures

Supplementary Figure 1. Detection of TraT on *Edwardsiella tarda*. *E. tarda* TX01 was incubated with anti-rTraT or anti-rTrx antibody and then with FITC-labeled secondary antibody. The cells were stained with DAPI and subjected to microscopy. Bar, 20 μm.

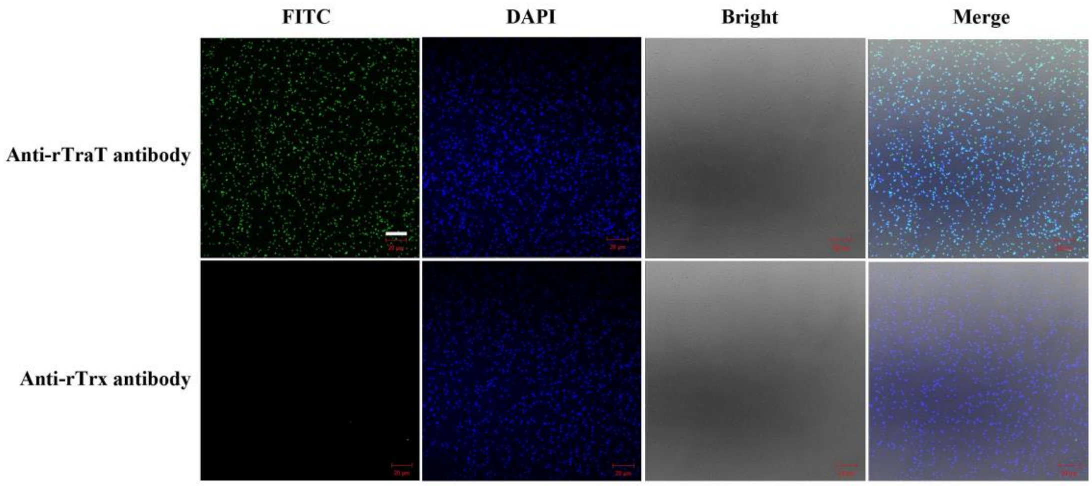

Supplementary Figure 2. rTraT – CFH interaction detected by surface plasmon resonance (SPR). Binding of CFH to rTraT (80 µg/ml, 40 µg/ml, 20 µg/ml, 10 µg/ml, 5 µg/ml, or 2.5 µg/ml), rTrx (80 µg/ml), or PBS (control) was analyzed by SPR.

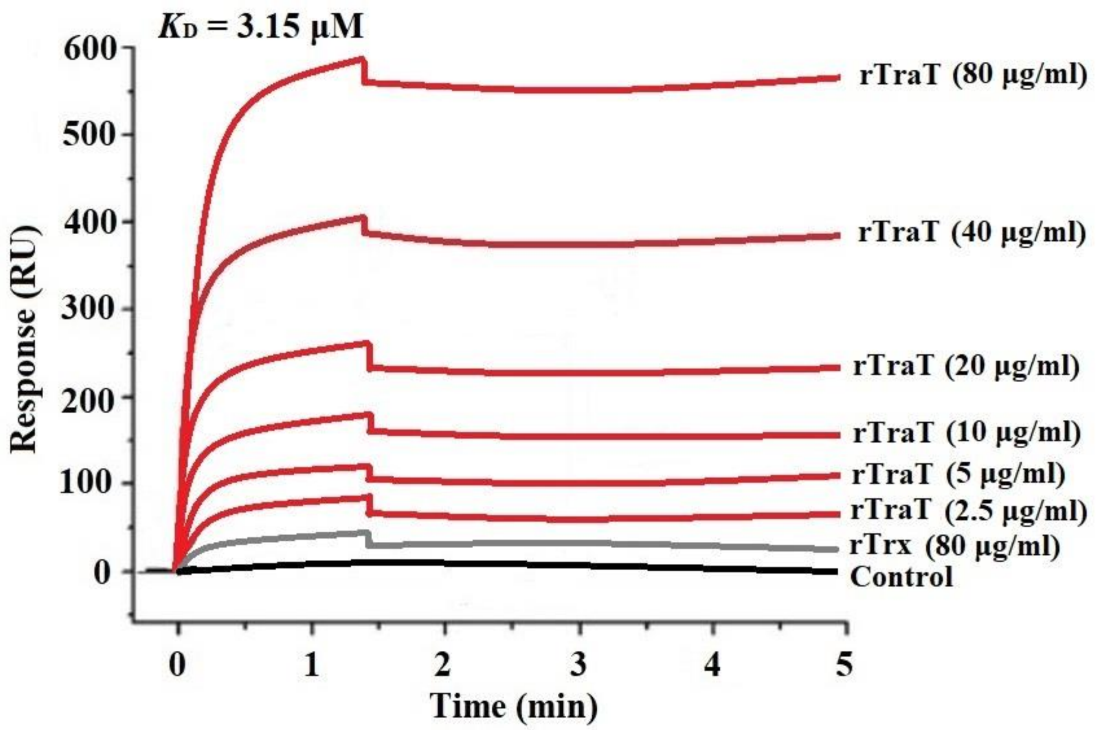

Supplementary Figure 3. Expression of TraT variants in *Escherichia coli*. *E. coli* expressing or not expressing (control) TraT wild type (WT) or mutants (M1 to M4) were analyzed by Western blot with anti-rTraT antibody. *Edwardsiella tarda* TX01 was included for comparison. Lane M, molecular weight markers.

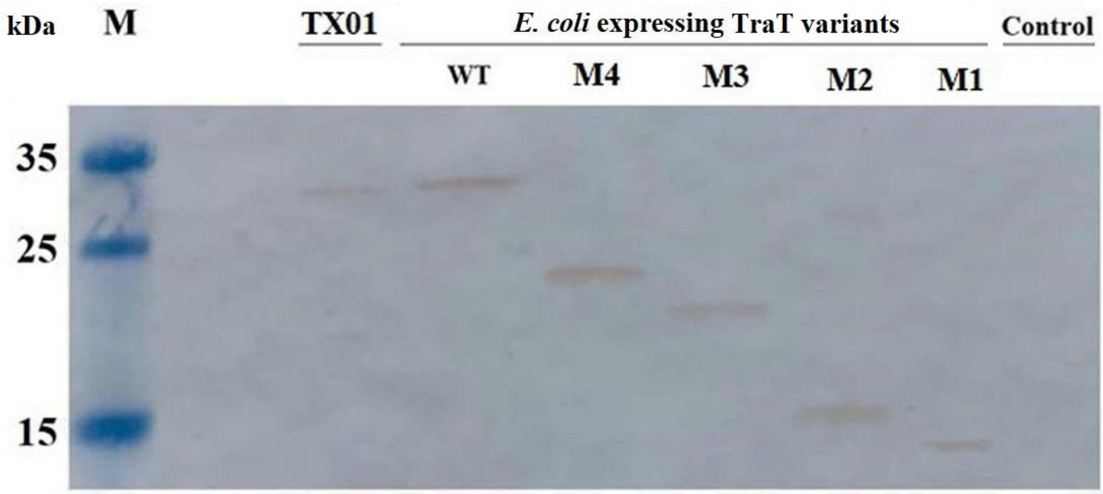

Supplementary Figure 4. The growth of *Edwardsiella tarda* variants under different conditions. *E. tarda* TX01 variants were cultured in LB medium (**a**) or LB medium supplemented with 1 mM H_2O_2 (**b**), and bacterial growth was monitored at different time points by measuring absorbance at 600 nm.

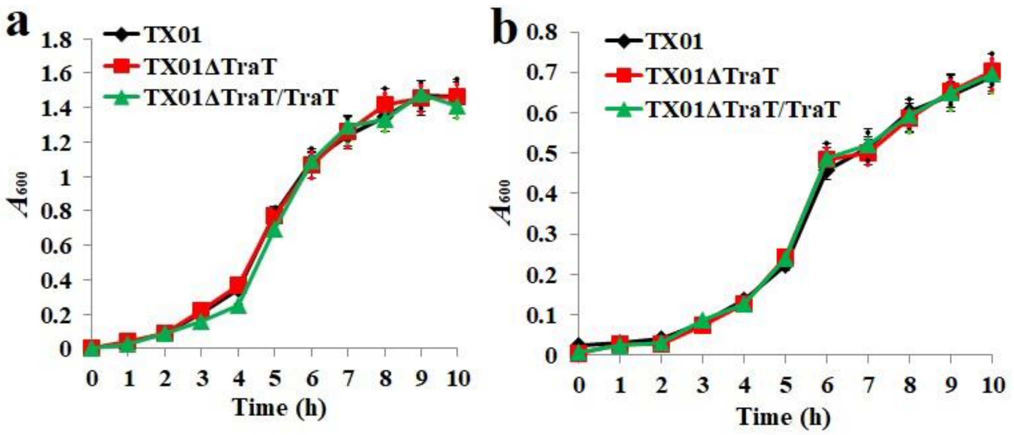

Supplementary Figure 5. Expression of Flag-tagged mCD46 in HEK293T cells. (**a**) HEK293T cells transfected with or without (control) the plasmid pmCD46Flag that expresses Flag-tagged mCD46 (mCD46-Flag-293T) or the backbone plasmid that expresses the Flag tag (Flag-293T) were treated with FITC-labeled antibody and stained with DAPI and Dil. Images of the cells were then taken. Bar, 20 μm. (**b**) The above transfected cells were subjected to Western blot with anti-Flag or anti-β-actin antibody.

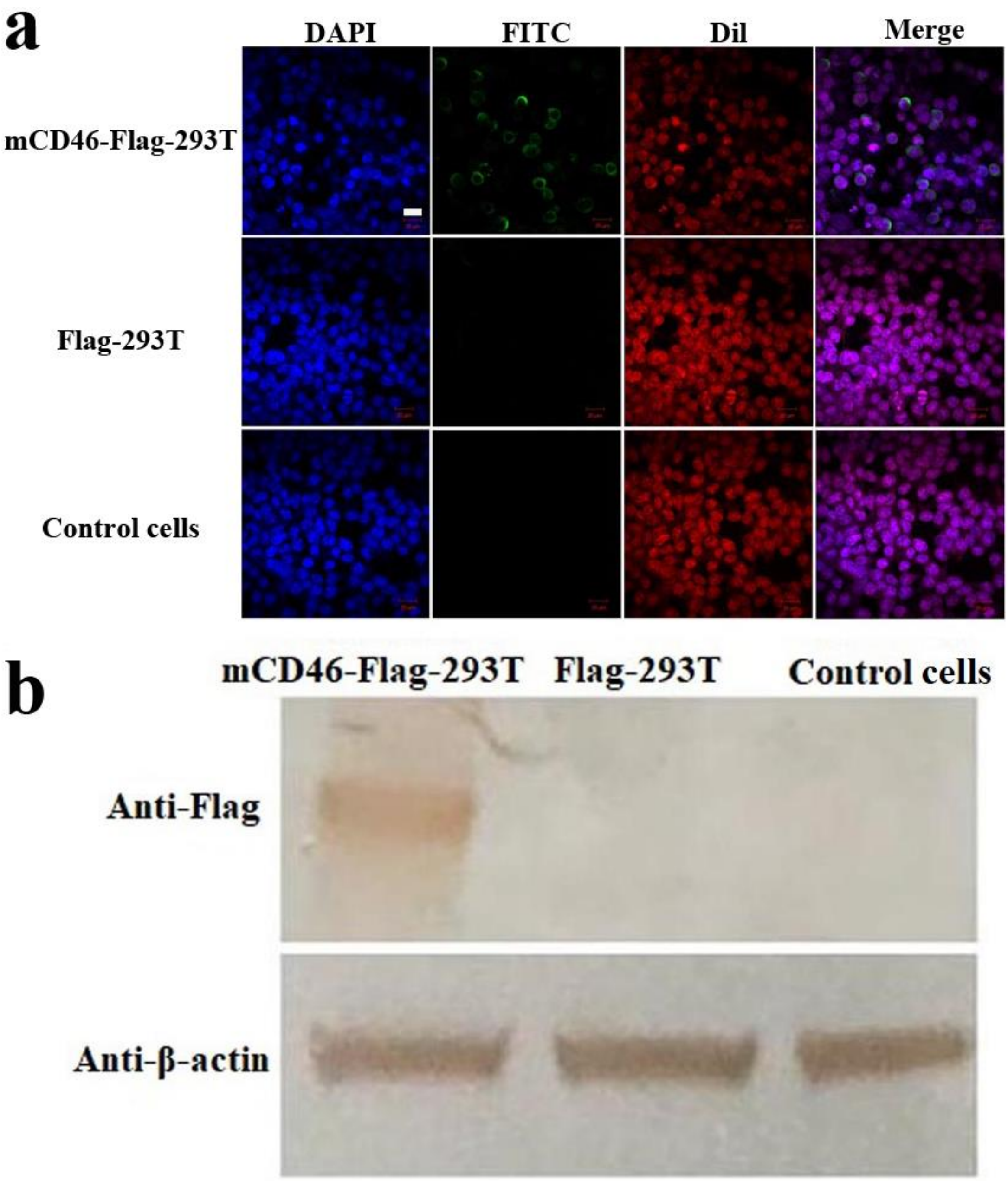

Supplementary Figure 6. Effect of rmCD46 on *Edwardsiella tarda* infection of RAW264.7 cells. RAW264.7 cells were infected with *E. tarda* TX01 that had been pre-incubated with or without (control) rmCD46 or rTrx for 1 h. The cells were treated with FITC-labeled antibody targeting TX01, and the bacteria binding was determined by measuring cell-bound fluorescence.

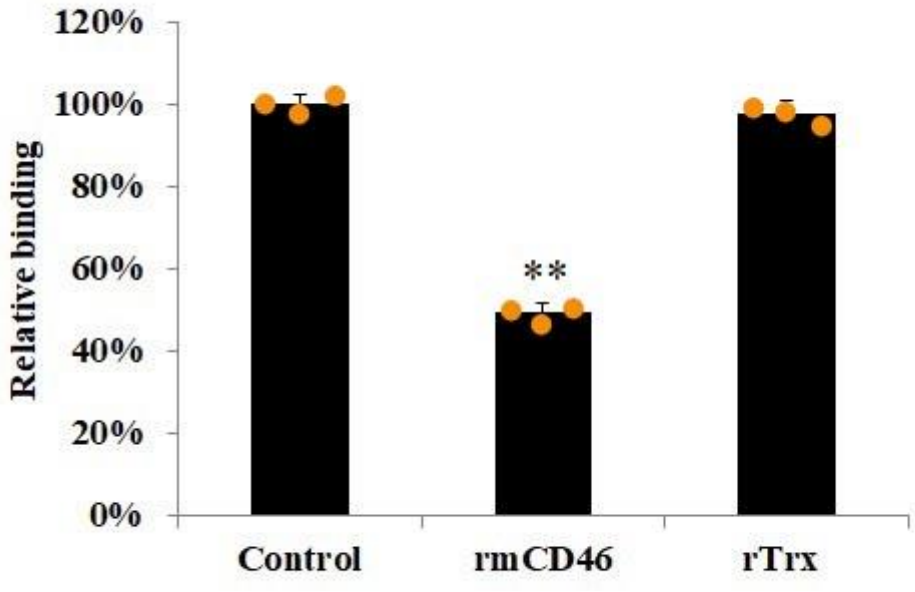

Supplementary Figure 7. The effect of *Edwardsiella tarda* on the binding of rmCD46 to C3b and CFI. rmCD46 was pre-incubated with or without *E. tarda* TX01 and then determined for C3b binding or CFI binding by ELISA. In all panels, the data are the means of three independent experiments and presented as means ±SEM.

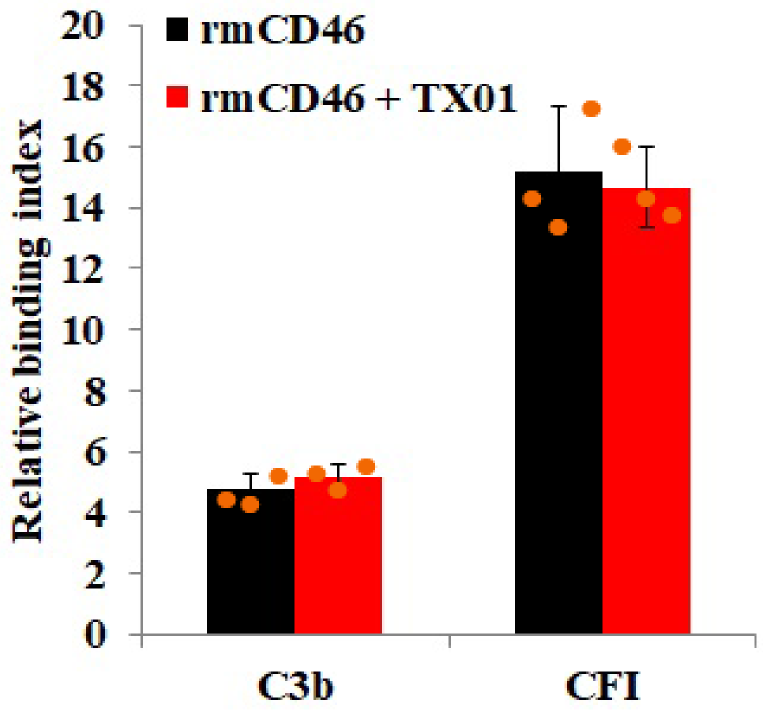

Supplementary Figure 8. Schematic presentation of the mCD46 variants. The domain structures of mCD46 (upper) and mCD46 mutants are schematically drawn. CCP, complement control protein.

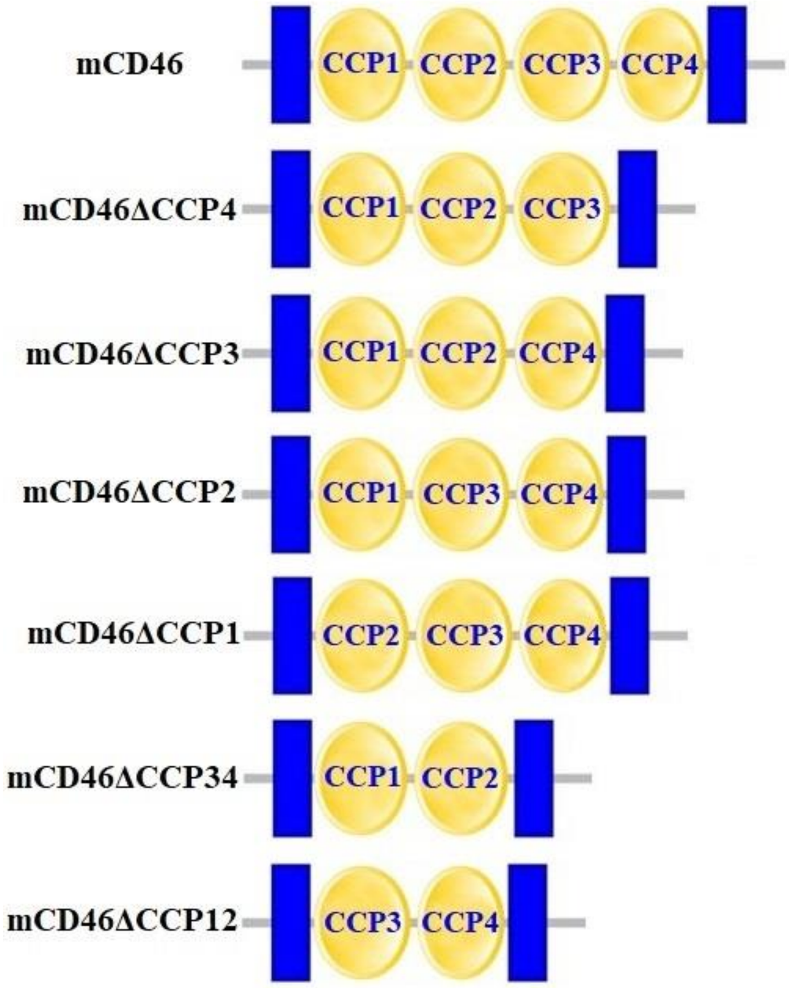

Supplementary Figure 9. Expression of mCD46 variants in HEK293T cells. HEK293T cells were transfected with the plasmid expressing Flag-tagged mCD46 wild type (WT) or mutants or the Flag alone. The cell lysate was subjected to Western blot with antibody against Flag or β-actin, which was used as a loading control. Lane M, molecular weight markers.

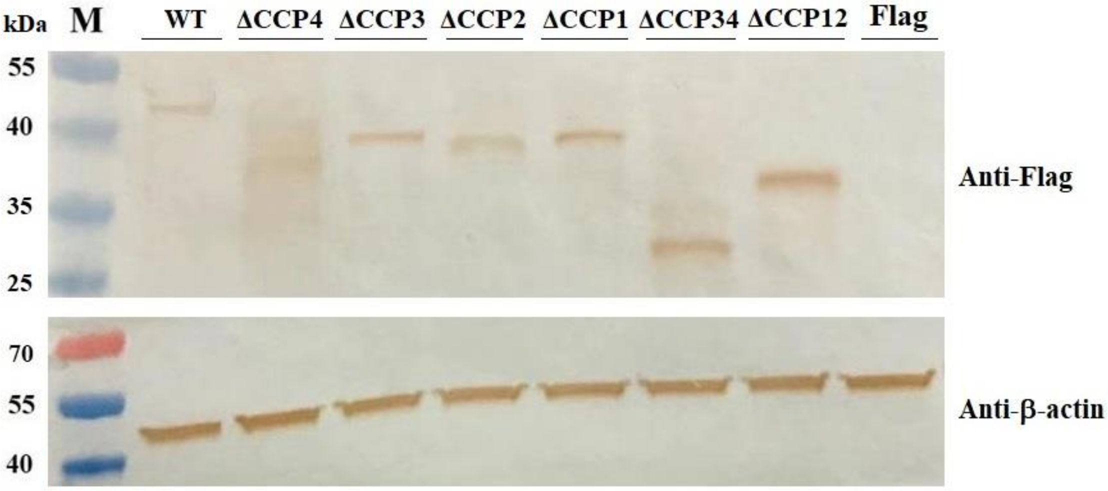

Supplementary Figure 10. The uncropped images of the Western blots of this study.

Figure 2a

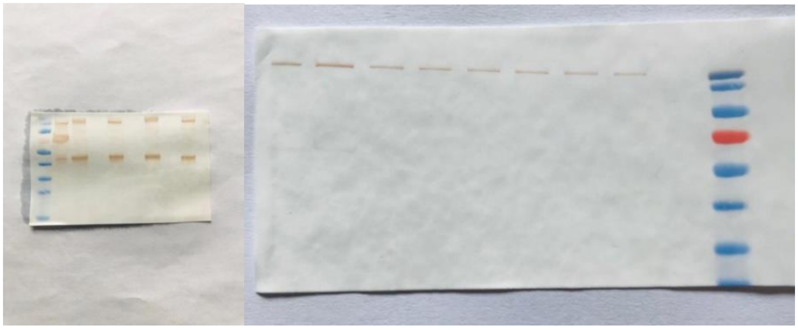

Figure 2b

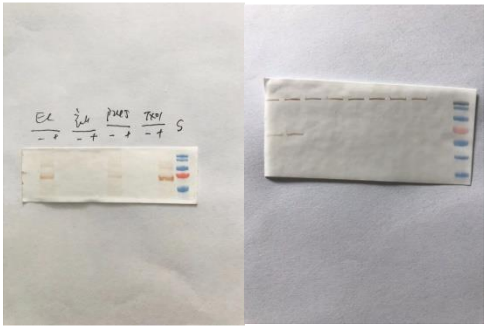

Figure 2c

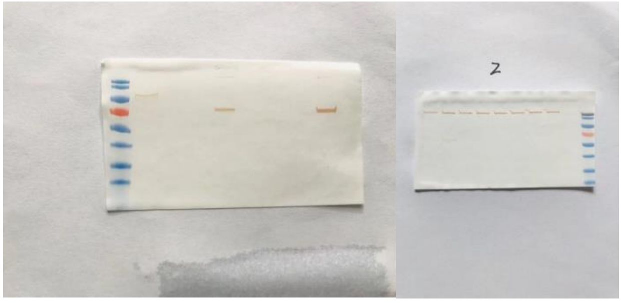

Figure 2d

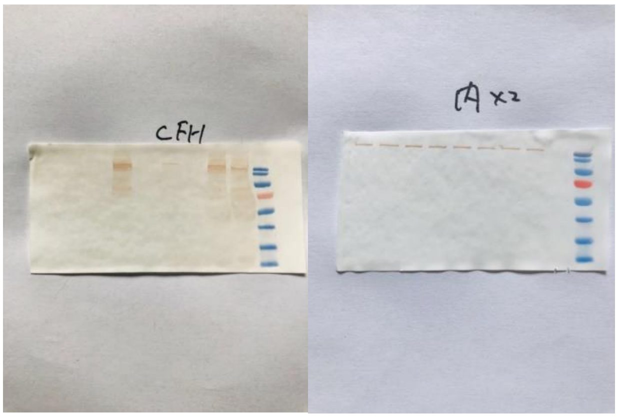

Figure 2e

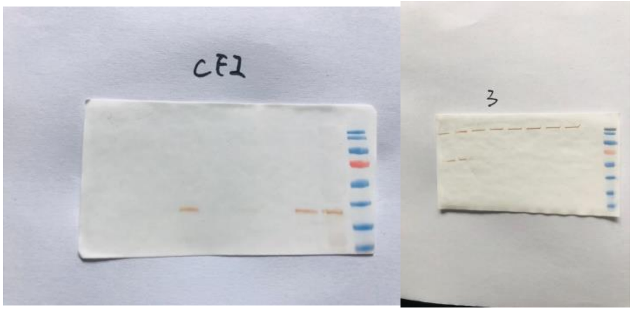

Figure 2g

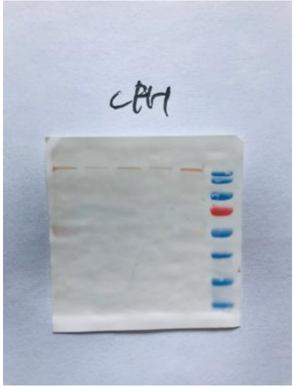

Figure 2h

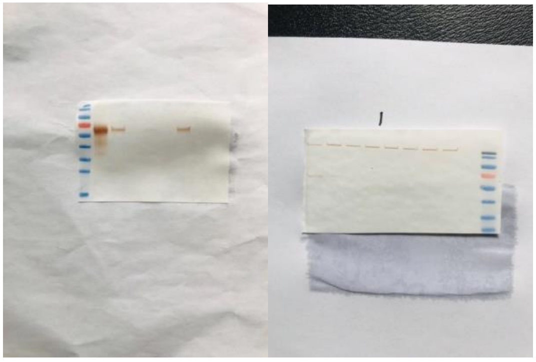

Figure 2j

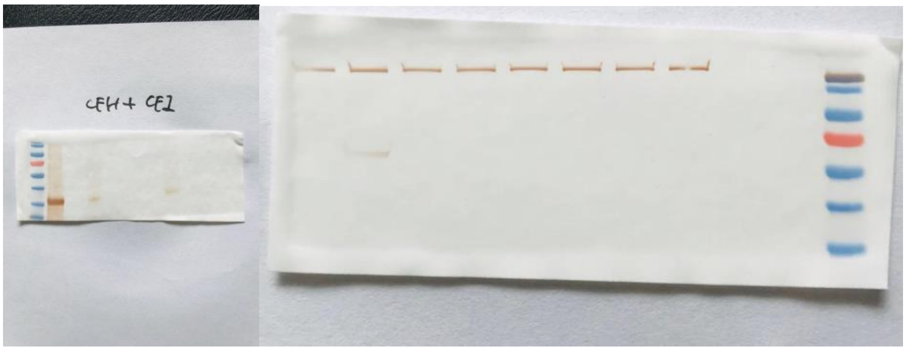

Figure 3a

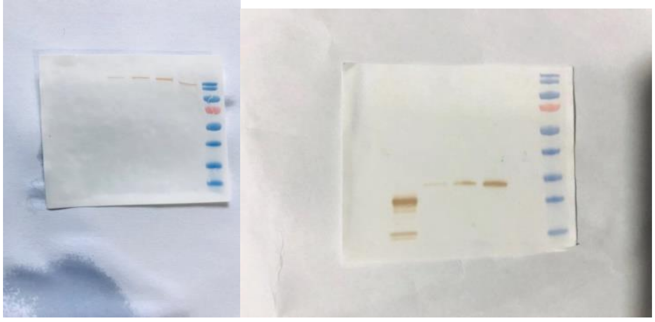

Figure 3g

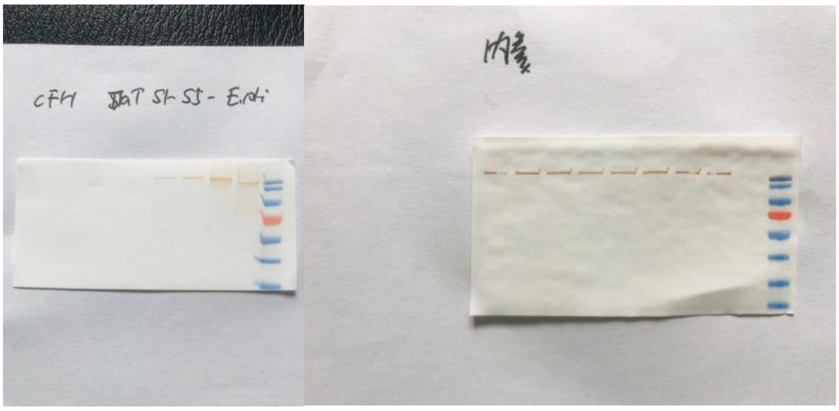

Figure 5d

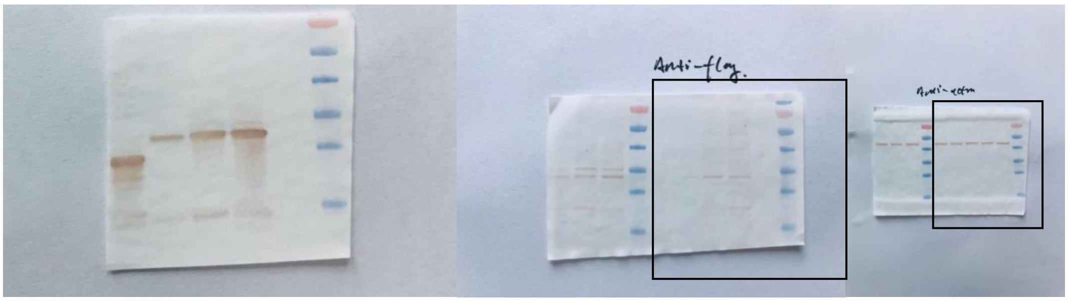

Supplementary Figure 3

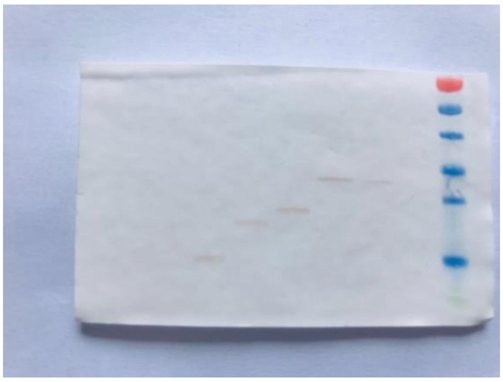

Supplementary Figure 5

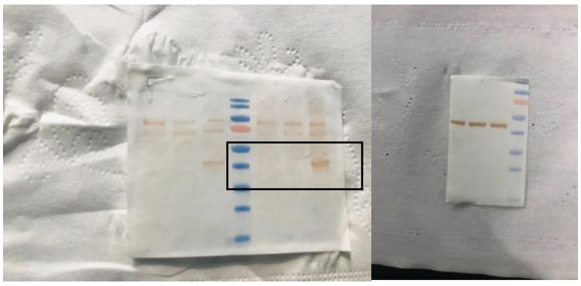

Supplementary Figure 9

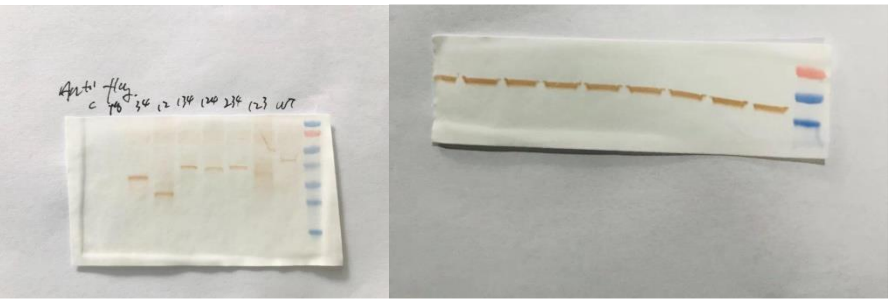

Supplementary tables

Supplementary Table 1. Bacterial strains and plasmids used in this study

Strains or plasmid	Source or reference	
Escherichia coli strains		
BL21 (DE3)	TransGen Biotech., Beijing, China	
DH5α	TransGen Biotech., Beijing, China	
S17-1 λ*pir*	Biomedal, Sevilla, Spain	
Edwardsiella tarda strains		
TX01	66,67	
TX01ΔtraT	This study	
TX01ΔtraT/traT	This study	
Plasmids		
T−A cloning vector T-Simple	TransGen Biotech., Beijing, China	
pET259	68	
pET28a	Sangon Biotech, Shanghai, China	
pET32a	Novagen, San Diego, USA	
pBT3	61	
pJT	69	
pDM4	70	
pCAGGS	MiaoLing Plasmid Platform, Wuhan, China	
pETTraT	This study	Addgene ID: 186783
pETmCD46	This study	Addgene ID: 186942
pCAGGSmCD46	This study	Addgene ID: 186943
pJTTraT	This study	Addgene ID: 186944
pJTTraTM1	This study	Addgene ID: 186945
pJTTraTM2	This study	Addgene ID: 186946
pJTTraTM3	This study	Addgene ID: 186947
pJTTraTM4	This study	Addgene ID: 186948
pDMTraT	This study	Addgene ID: 186949

Supplementary Table 2. Primers used in this study

Primer	Sequence (5’-3’)[a]
TraT-F	GATATCATGATTAAGAAGCGTAATCTGGAGG (*Eco*RV)
TraT-R	GATATCCAGAATGCTGGCGATGGATT (*Eco*RV)
TraT-F1	GATATCATGAACCGCCATTATAAAAATG (*Eco*RV)
TraT-R1	GATATCCAGAATGCTGGCGATGGATT (*Eco*RV)
TraT-MR1	GATATCGGGGTTGGAGACCAGCTGAT (*Eco*RV)
TraT-MR2	GATATCCGCGGCCCCCGTGGCCGCCC (*Eco*RV)
TraT-MR3	GATATCGACATCCTCGACCATCGCAT (*Eco*RV)
TraT-MR4	GATATCATGCTGGTTACCCTGCATGTTG (*Eco*RV)
TraT-F2	GGATCCGTAACCCGTGTTGCCAGAA (*Bam*HI)
TraT-R2	TTGCTGGTCAGCAGCAGACAAAAGGAGA
TraT-F3	TGCTGCTGTCCATCGCCAGCATTCTGTA
TraT-R3	GGATCCGCGGAAACGGTTCTGGTCTT (*Bam*HI)
mCD46-F	CCCGGGATGGATGCCTGTGAACTACCACG (*Sma*I)
mCD46-R	CCCGGGTGCATCTAATTCTTGGCTAA (*Sma*I)
mCD46-F1	GAGCTCATGACGGCGGCGCCTCTTAT (*Sca*I)
mCD46-R1	GCTAGCTCATCTTGCTGCAGATACAT (*Nhe*I)
TNFα-RT-F	CCACATCTCCCTCCAGAAAA
TNFα-RT-R	AGGGTCTGGGCCATAGAACT
IL1β-RT-F	AGTTGACGGACCCCAAAAG
IL1β-RT-R	AGCTGGATGCTCTCATCAGG
IL6-RT-F	GACAAAGCCAGAGTCCTTCAGAGAG
IL6-RT-R	CTAGGTTTGCCGAGTGATCTC
IL18-RT-F	GTAAGAGGACTGGCTGTGACCC
IL18-RT-R	CTTTTGGCAAGCAAGAAAGTGT
IL27-RT-F	CTTCCCAATGTTTCCCTGAC
IL27-RT-R	CGAAGTGTGGTAGCGAGGA
IL33-RT-F	CCTGCCTCCCTGAGTACATACA
IL33-RT-R	CGGAGTAGTCCTTGTCGTTGG
CXCL2-RT-F	TCAAGAACATCCAGAGCTTGAGT
CXCL2-RT-R	AGCCTTGCCTTTGTTCAGTATC

[a]Underlined nucleotides are restriction sites of the enzymes indicated in the parentheses at the end of the sequence.
