# Supplementary material for: Edwardsiella tarda TraT is an anti-complement factor and a cellular infection promoter
Source: Commun Biol. 2022 Jun 29;5:637. doi: 10.1038/s42003-022-03587-3 (PMC9243006; doi:10.1038/s42003-022-03587-3)
Supplement: Supplementary file 3 — Description of Additional Supplementary Files [file 42003_2022_3587_MOESM3_ESM.pdf]

Description of Additional Supplementary Files

File name: Supplementary Data 1
Description: The source data in this study.
