# Supplementary material for: Edwardsiella tarda TraT is an anti-complement factor and a cellular infection promoter
Source: Commun Biol. 2022 Jun 29;5:637. doi: 10.1038/s42003-022-03587-3 (PMC9243006; doi:10.1038/s42003-022-03587-3)
Supplement: Supplementary file 5 — Reporting Summary [file 42003_2022_3587_MOESM5_ESM.pdf]

nature portfolio

Corresponding author(s): Li Sun

Last updated by author(s): Apr 29, 2022

Reporting Summary

Nature Portfolio wishes to improve the reproducibility of the work that we publish. This form provides structure for consistency and transparency in reporting. For further information on Nature Portfolio policies, see our Editorial Policies and the Editorial Policy Checklist.

Statistics

For all statistical analyses, confirm that the following items are present in the figure legend, table legend, main text, or Methods section.

n/a	Confirmed	
☐	☒	The exact sample size (*n*) for each experimental group/condition, given as a discrete number and unit of measurement
☐	☒	A statement on whether measurements were taken from distinct samples or whether the same sample was measured repeatedly
☐	☒	The statistical test(s) used AND whether they are one- or two-sided<br>*Only common tests should be described solely by name; describe more complex techniques in the Methods section.*
☒	☐	A description of all covariates tested
☒	☐	A description of any assumptions or corrections, such as tests of normality and adjustment for multiple comparisons
☐	☒	A full description of the statistical parameters including central tendency (e.g. means) or other basic estimates (e.g. regression coefficient) AND variation (e.g. standard deviation) or associated estimates of uncertainty (e.g. confidence intervals)
☒	☐	For null hypothesis testing, the test statistic (e.g. *F*, *t*, *r*) with confidence intervals, effect sizes, degrees of freedom and *P* value noted<br>*Give P values as exact values whenever suitable.*
☒	☐	For Bayesian analysis, information on the choice of priors and Markov chain Monte Carlo settings
☒	☐	For hierarchical and complex designs, identification of the appropriate level for tests and full reporting of outcomes
☒	☐	Estimates of effect sizes (e.g. Cohen's *d*, Pearson's *r*), indicating how they were calculated

Our web collection on statistics for biologists contains articles on many of the points above.

Software and code

Policy information about availability of computer code

Data collection	The sequences of TraT and CD46 were directly downloaded from NCBI (www.ncbi.nlm.nih.gov) .
Data analysis	Sequence analysis was performed using the BLAST program at the National Center for Biotechnology Information (NCBI) and the Expert Protein Analysis System. Domain search was performed with the conserved domain search program of NCBI and SMART. Theoretical molecular mass and isoelectric point (pI) were predicted using Expasy compute PI/Mw tool. Multiple sequence alignment was created with DNAMAN. Subcellular localization prediction was performed with CELLO v.2.5 (http://cello.life.nctu.edu.tw/). The 3D structural figures were generated using I-TASSER of Zhang Lab (https://zhanggroup.org/I-TASSER/).

For manuscripts utilizing custom algorithms or software that are central to the research but not yet described in published literature, software must be made available to editors and reviewers. We strongly encourage code deposition in a community repository (e.g. GitHub). See the Nature Portfolio guidelines for submitting code & software for further information.

Data

Policy information about availability of data

All manuscripts must include a data availability statement. This statement should provide the following information, where applicable:

- Accession codes, unique identifiers, or web links for publicly available datasets
- A description of any restrictions on data availability
- For clinical datasets or third party data, please ensure that the statement adheres to our policy

The TraT sequence of Edwardsiella tarda is from NCBI, and its GenBank accession number is WP_015461079.1. The CD46 sequence of Mus musculus is from NCBI, and its GenBank accession number is BAA31859.1.

Field-specific reporting

Please select the one below that is the best fit for your research. If you are not sure, read the appropriate sections before making your selection.

☒ Life sciences ☐ Behavioural & social sciences ☐ Ecological, evolutionary & environmental sciences

For a reference copy of the document with all sections, see nature.com/documents/nr-reporting-summary-flat.pdf

Life sciences study design

All studies must disclose on these points even when the disclosure is negative.

Sample size	In animal study, 3 mice were used per time point to examine bacterial dissemination in tissues, and 10 mice were used in each group for mortality analysis.
Data exclusions	No data were excluded.
Replication	All attempts at replication were successful.
Randomization	The animals were randomly selected or randomly allocated into experimental groups.
Blinding	The investigators were not blinded to group allocation.

Reporting for specific materials, systems and methods

We require information from authors about some types of materials, experimental systems and methods used in many studies. Here, indicate whether each material, system or method listed is relevant to your study. If you are not sure if a list item applies to your research, read the appropriate section before selecting a response.

Materials & experimental systems

n/a	Involved in the study	
☐	☒	Antibodies
☐	☒	Eukaryotic cell lines
☒	☐	Palaeontology and archaeology
☐	☒	Animals and other organisms
☒	☐	Human research participants
☒	☐	Clinical data
☒	☐	Dual use research of concern

Methods

n/a	Involved in the study	
☒	☐	ChIP-seq
☐	☒	Flow cytometry
☒	☐	MRI-based neuroimaging

Antibodies

Antibodies used

Rabbit monoclonal to C3 (Abcam, Cambridge, MA, USA) (CAT# ab200999)
Rabbit monoclonal to C5 (Abcam, Cambridge, MA, USA) (CAT# ab275931)
Rabbit monoclonal to complement factor B (Abcam, Cambridge, MA, USA) (CAT# ab133765)
Sheep polyclonal to complement factor H (Abcam, Cambridge, MA, USA) (CAT# ab8842)
Anti-RNA polymerase beta antibody (Abcam, Cambridge, MA, USA) (CAT# Ab191598)
Rabbit polyclonal to factor I (Abcam, Cambridge, MA, USA) (CAT# ab231969)
Rabbit polyclonal to CD46 (Abcam, Cambridge, MA, USA) (CAT# ab135397)
HRP goat anti-rat IgG (H + L) antibody (Abcam, Cambridge, MA, USA) (CAT# ab205720)
HRP goat anti-mouse IgG (H + L) antibody (Abcam, Cambridge, MA, USA) (CAT# ab205719)
HRP goat anti-rabbit IgG (H + L) antibody (Abconal, Wuhan, China) (CAT# AS014)
HRP rabbit anti-Sheep IgG (H + L) antibody (Abconal, Wuhan, China) (CAT# AS023)
HRP-conjugated Mouse anti Flag-tag (Abconal, Wuhan, China) (CAT# AE024)
Mouse anti-Flag antibody (Abconal, Wuhan, China) (CAT# AE005)
Mouse anti-His antibody (Abconal, Wuhan, China) (CAT# AE003)
Alexa Fluor 594 goat anti-mouse IgG (H + L) antibody (Abcam, Cambridge, MA, USA) (CAT# Ab150120)
FITC goat anti-mouse IgG (H + L) antibody (Abcam, Cambridge, MA, USA) (CAT# Ab150117)
Mouse polyclonal antibodies against rTraT and rTrx were prepared in this study.

Validation

The commercial antibodies were validated by the manufacturers. The antibodies prepared in this study were validated by ELISA and Western blot analyses.

Eukaryotic cell lines

Policy information about cell lines

Cell line source(s)	RAW264.7 and HEK293T cell lines were purchased from Cell Resource Center, Beijing, China.
Authentication	The cell lines were commercial and purchased from Cell Resource Center, Beijing, China.
Mycoplasma contamination	No Mycoplasma contamination.
Commonly misidentified lines (See ICLAC register)	No commonly misidentified cell lines were used in this study.

Animals and other organisms

Policy information about studies involving animals; ARRIVE guidelines recommended for reporting animal research

Laboratory animals	Mus musculus, C57BL/6, adult, female, 6-8 week.
Wild animals	No wild animals were used in this study.
Field-collected samples	The study did not involve samples collected from the field.
Ethics oversight	The experiments involving live animals conducted in this study were approved by the Ethics Committee of Institute of Oceanology, Chinese Academy of Sciences.

Note that full information on the approval of the study protocol must also be provided in the manuscript.

Flow Cytometry

Plots

Confirm that:

☒ The axis labels state the marker and fluorochrome used (e.g. CD4-FITC).

☒ The axis scales are clearly visible. Include numbers along axes only for bottom left plot of group (a 'group' is an analysis of identical markers).

☒ All plots are contour plots with outliers or pseudocolor plots.

☒ A numerical value for number of cells or percentage (with statistics) is provided.

Methodology

Sample preparation	To determine the effect of CFH on the cellular binding of rTraT, rTraT (40 μg/ml) was incubated with or without CFH (20 μg/ml), rTrx (20 μg/ml), or PBS for 2 h at 28 ?. The mixture was then incubated with RAW264.7 cells for 2 h at 28 ? and then washed three times with PBS. Mouse anti-His antibody and FITC-labeled goat anti-mouse IgG were added into the cells. The cells were washed with PBS and analyzed by flow cytometry with a FACSAria II flow cytometer.
Instrument	BD FACSAria II
Software	BD FACSDiva3.0
Cell population abundance	1×10 6
Gating strategy	SSC×FSC include RAW264.7 cells, and FITC-stained cells were defined as positive cells.

☐ Tick this box to confirm that a figure exemplifying the gating strategy is provided in the Supplementary Information.
